# Oncolytic Adenovirus Expressing IL-23 and p35 Elicits IFN-γ- and TNF-α-Co-Producing T Cell-Mediated Antitumor Immunity

**DOI:** 10.1371/journal.pone.0067512

**Published:** 2013-07-03

**Authors:** Il-Kyu Choi, Yan Li, Eonju Oh, Jaesung Kim, Chae-Ok Yun

**Affiliations:** 1 Department of Bioengineering, College of Engineering, Hanyang University, Seoul, Korea; 2 Graduate Program for Nanomedical Science, Yonsei University, Seoul, Korea; 3 Department of Pharmaceutics and Pharmaceutical Chemistry, Center for Controlled Chemical Delivery, University of Utah, Salt Lake City, Utah, United States of America; The University of Chicago, United States of America

## Abstract

Cytokine immunogene therapy is a promising strategy for cancer treatment. Interleukin (IL)-12 boosts potent antitumor immunity by inducing T helper 1 cell differentiation and stimulating cytotoxic T lymphocyte and natural killer cell cytotoxicity. IL-23 has been proposed to have similar but not overlapping functions with IL-12 in inducing Th1 cell differentiation and antitumor immunity. However, the therapeutic effects of intratumoral co-expression of IL-12 and IL-23 in a cancer model have yet to be investigated. Therefore, we investigated for the first time an effective cancer immunogene therapy of syngeneic tumors via intratumoral inoculation of oncolytic adenovirus co-expressing IL-23 and p35, RdB/IL23/p35. Intratumoral administration of RdB/IL23/p35 elicited strong antitumor effects and increased survival in a murine B16-F10 syngeneic tumor model. The levels of IL-12, IL-23, interferon-γ (IFN-γ), and tumor necrosis factor-α (TNF-α) were elevated in RdB/IL23/p35-treated tumors. Moreover, the proportion of regulatory T cells was markedly decreased in mice treated with RdB/IL23/p35. Consistent with these data, mice injected with RdB/IL23/p35 showed massive infiltration of CD4^+^ and CD8^+^ T cells into the tumor as well as enhanced induction of tumor-specific immunity. Importantly, therapeutic mechanism of antitumor immunity mediated by RdB/IL23/p35 is associated with the generation and recruitment of IFN-γ- and TNF-α-co-producing T cells in tumor microenvironment. These results provide a new insight into therapeutic mechanisms of IL-12 plus IL-23 and provide a potential clinical cancer immunotherapeutic agent for improved antitumor immunity.

## Introduction

Despite many immunologists have intensively studied to eradicate a cancer during the last decade, the cancer still remained resistant to conventional immunotherapy due to various immune evasion mechanisms mediated by tumors [1]–[3]. In other words, the cancer made efforts to generate more favorable tumor microenvironment for cancer development, spread, and metastasis. Hence, therapeutic efficacy might be improved by effective methodologies that have focused on overcoming tumor-induced immune suppression and generating enhanced antitumor immune response.

Immunogene therapy is one of the cancer immunotherapeutic strategies that involve the delivery of immune genes to induce the antitumor adaptive immunity in the tumor milieu. Many immune stimulatory factors have been used in cancer immunogene therapy [4], [5]. In particular, cancer immunogene therapy using cytokine genes might represent further advancement in the cancer therapeutics, since it has a great potential for identifying and eradicating cancer cells by activating tumor-specific immune responses in cancer-bearing hosts [4], [6]. Moreover, cytokine gene-based cancer immunotherapy can suppress the metastasis and recurrence of the cancer through the generation of a tumor-specific immunologic memory [7].

Interleukin (IL)-12 has demonstrated to be one of the most effective and promising antitumor cytokine. It is a heterodimeric cytokine composed of two different disulfide-linked subunits designated p35 and p40, and when coordinately expressed within one cell, biologically active IL-12 is produced. IL-12 stimulates interferon-γ (IFN-γ) and tumor necrosis factor-αTNF-αproduction by natural killer (NK) cells and T cells, eliciting promoted the T helper 1 (Th1) immune response [8], [9]. Previous preclinical studies of IL-12 have been shown to exert significant antitumor immunity in various murine tumor models [10]. More recently, IL-12-based clinical trials have been performed with human cancer-bearing patients [11], [12]. However, objective clinical benefits were fewer than expected. The repeated intratumoral administration of IL-12 leaded to several potential immunosuppressive mechanisms that were associated with the polarization from a Th1 to Th2 immunity as illustrated by an elevation in IL-10 expression and decrease of IFN-γ and TNF-α in the sera of patients repeatedly received with IL-12 [11], [12]. These clinical outcomes indicate a potential limitation in the use of IL-12 as a single agent for the treatment of cancer. Therefore, IL-12-mediated antitumor efficacy may be enhanced by the addition of an adjuvant to overcome immunosuppressive microenvironments induced by tumors and to induce optimally differentiated tumor-specific T cells.

IL-23 is a covalently linked heterodimeric cytokine that comprises of a novel p19 subunit which is structurally related to the p35 subunit of IL-12 and the p40 subunit of IL-12 [13]. It also needs co-expression of both p19 and p40 subunits within the identical cell to form the biologically active IL-23 molecule. IL-23, like IL-12, is secreted by activated macrophages and DCs. In addition, IL-23 has been shown to have significant antitumor effects in various establishment models of cancer, making it an important candidate for cancer immunogene therapy [14], [15]. These studies indicate that the therapeutic mechanism mediated by IL-23 is associated with the promotion of cell-mediated immune response and activation of CTLs or NK cells, similar to IL-12. These similar functional and structural characteristics of IL-12 and IL-23 suggest that IL-23, acting in concert with IL-12, may have considerable promise for effective cytokine gene-based cancer immunotherapy. This suggestion is further supported by the finding that endogenous IL-12 in the cancer-bearing host made a significant contribution to IL-23-mediated anti-tumor immunity [14]. However, the effects of local, intratumoral co-expression of IL-12 and IL-23 in a therapeutic cancer model have yet to be investigated.

In the present study, we show for the first time an effective cancer immunogene therapy of syngeneic tumors via intratumoral administration of oncolytic adenovirus (Ad) co-expressing IL-23 and p35. The oncolytic Ad co-expressing IL-23 and p35 elicited potent antitumor effect, correlated with up-regulation of IL-12, IL-23, IFN-γ, and TNF-αwithin the tumor tissues as well as the significant reduction of regulatory T (Treg) cell frequency. Moreover, we demonstrate therapeutic mechanism of antitumor immune response mediated by oncolytic Ad co-expressing IL-23 and p35, showing that antitumor immunity is associated with the generation and recruitment of IFN-γ- and TNF-α-co-producing T cells in tumor microenvironment.

## Results

### Enhanced expression of both IL-12 and IL-23 by oncolytic Ad single vector expressing IL-23 and p35

**Figure 1:**
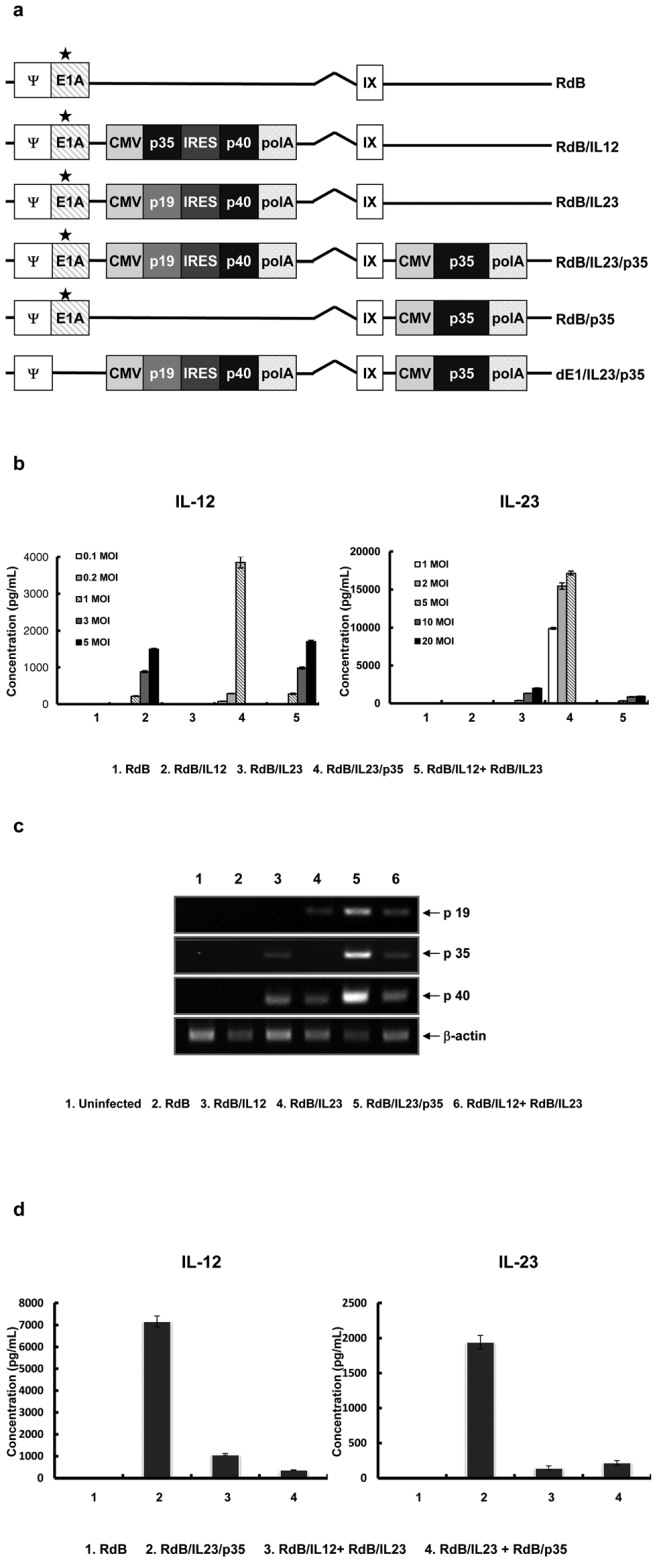
Characterization of Ad vectors. (a) Schematic representation of the genomic structures of Ads. RdB contains mutated E1A, but lacks E1B 19 and 55 kD, and E3 region. RdB/IL12 and RdB/IL23 are comprised of the IL-12 or IL-23 genes in the E1 region of RdB, respectively. RdB/IL23/p35 is comprised of the IL-23 and p35 genes in the E1 and E3 region of RdB, respectively (mutation at Rb binding site of E1A). RdB/p35 is comprised of the p35 genes in the E3 region of RdB. dE1/IL23/p35 is comprised of the IL-23 and p35 genes in the E1 and E3 region of dE1 (replication-incompetent Ad), respectively. Translational (b) and transcriptional (c) level of IL-12 and IL-23 were assessed in U343 cells after infection with Ads at different MOIs. Cell culture supernatants or cells were harvested at 48 hr after infection, and the level of IL-12 and IL-23 was quantified by conventional ELISA kit or RT-PCR assay. ELISA data represent the mean ± SE of triplicate experiments. (d) Up-regulation of IL-12 and IL-23 induced by RdB/IL23/p35 regardless of p40 gene dose. Level of IL-12 and IL-23 expression was assessed in U343 cells at 48 hr after infection. The concentration of cytokines was measured in the culture supernatants by ELISA kit. Data represent the mean ± SE of triplicate experiments.

Ad has been demonstrated to accommodate up 105% of the wild-type genome, and Ad vector with genome larger than this capacity leads to nonviable or extremely unstable, frequently undergoing DNA rearrangements [16]. Accordingly, to get over the size limitation of Ad genome, we generated an oncolytic Ad co-expressing IL-23 and IL-12 (RdB/IL23/p35) by inserting IL-23 gene (p19, IRES, and p40) in E1 region and p35 gene in the E3 region, instead of inserting IL-23 gene (p19, IRES, and p40) and IL-12 gene (p35, IRES, and p40) in the E1 and E3 region, respectively (Figure 1a). Since p40 is shared by both IL-12 and IL-23 genes, we expected to express both IL-12 and IL-23 genes by utilizing p35 gene and full sequence of IL-23 gene which is composed of p19 and p40. To examine the level of IL-12 and IL-23 expression from RdB/IL23/p35 oncolytic Ad, U343 cells were infected with RdB/IL12, RdB/IL23, or RdB/IL23/p35 at different MOIs. Two days following transduction, IL-12 and IL-23 ELISA were carried out on cell supernatants. The results showed a significantly increased expression levels of the virally-transduced IL-12 or IL-23, whose level increased in a MOI-dependent manner (Figure 1b). More specifically, the IL-12 secretion level by RdB/IL23/p35 infection at 1 MOI was 3854±155 pg/mL, whereas that by RdB/IL12 and RdB/IL12 plus RdB/IL23 was 216±9 pg/mL and 279±16 pg/mL, respectively. Similarly, the IL-23 secretion level by RdB/IL23/p35 infection at 5 MOI was 17155±258 pg/mL, while that by RdB/IL23 and RdB/IL12 plus RdB/IL23 was 376±3 pg/mL and 327±31 pg/mL, respectively. Of note, these data show that RdB/IL23/p35 induces dramatically enhanced IL-12 and IL-23 secretion level by 18-fold and 46-fold, respectively compared with oncolytic Ad expressing RdB/IL12 alone or RdB/IL23 alone. Moreover, RdB/IL23/p35 elicited much higher levels of both IL-12 and IL-23 by 14-fold and 53-fold, respectively compared with RdBIL12 plus RdBIL23, implying that single vector (RdB/IL23/p35) has better efficiency than double vector (RdBIL12 plus RdBIL23) in terms of gene transfer efficiency.

To investigate whether the elevated mRNA level is responsible for enhanced expression levels of IL-12 and Il-23 mediated by RdB/IL23/p35, mRNA levels of p19, p35, and p40 were assessed after infecting U343 cells with RdB/IL12, RdB/IL23, RdB/IL23/p35, or RdB/IL12 plus RdB/IL23 at an MOI of 5. As seen in Figure 1c, mRNA levels of p19, p35, and p40 were significantly increased in the cells infected with RdB/IL23/p35 compared with those levels in the cells infected with RdB/IL12, RdB/IL23, or RdB/IL12 plus RdB/IL23, suggesting that single oncolytic Ad vector harboring IL-23 and p35 shows enhanced gene expression at transcriptional level as well as translational level compared with oncolytic Ad expressing IL-12 alone, IL-23 alone, or double oncolytic Ad vector (RdBIL12 plus RdBIL23).

### Up-regulation of IL-12 and IL-23 induced by RdB/IL23/p35 is not influenced by p40 gene dose

Previous reports have been demonstrated that homodimer of p40 can act as an antagonist of IL-12 and IL-23 biological activity [17], [18]. Hence, we further examined if gene dose of p40 affects the observed elevated expression levels of IL-12 and IL-23 mediated by RdB/IL23/p35. After generating an oncolytic Ad expressing only p35 (RdB/p35), we infected U343 cells together with RdB/IL23 to make equal gene dose of RdB/IL23/p35. As shown in Figure 1d, the expression level of IL-12 and IL-23 mediated by RdB/IL23/p35 infection was notably higher by 20-fold and 9-fold, respectively than those mediated by RdB/IL23 plus RdB/p35 infection, implying that high IL-12 and IL-23 expression levels expressed by RdB/IL23/p35 is not because of different p40 gene dose from double oncolytic Ad vectors which have double gene copy numbers of p40.

### Potent antitumor effect of IL-23- and p35-co-expressing oncolytic Ad

**Figure 2:**
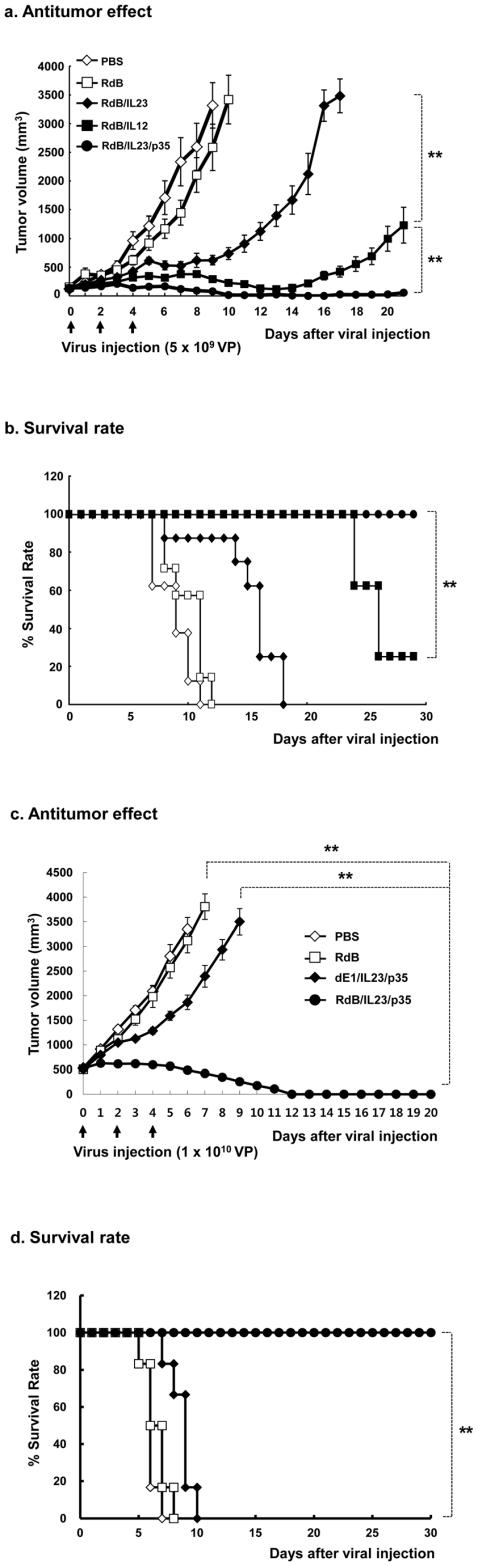
Antitumor effect and survival rate of tumor-bearing mice. Antitumor effect (a and c) and survival rate (b and d) in mice given PBS (◊), RdB (□), RdB/IL23 or dE1/IL23/p35 (♦), RdB/IL12 (▪), or RdB/IL23/p35 (•). C57BL/6 tumor-bearing mice were treated with intratumoral injections of 5×10^9^ VP/50 μL (a and b; initial tumor volume: 130-150 mm^3^) or 1×10^10^ VP/150 μL (c and d; initial tumor volume: 500 mm^3^) of Ads on days 0, 2, and 4. Tumor volume was monitored and recorded every day until the end of the study. The arrow represents Ad inoculation. Values represent the mean ± SE (6∼8 animals per group). **, P<0.01.

To assess the therapeutic efficacy of locally delivered RdB/IL23/p35 *in vivo* in comparison to oncolytic Ad expressing IL-12 alone or IL-23, B16-F10 melanoma tumors established in C57BL/6 mice were treated with RdB/IL12, RdB/IL23, or RdB/IL23/p35, along with control oncolytic Ad, RdB by direct intratumoral injection. By 11 days post-treatment, all mice in the PBS control group become not viable because of robust tumor growth (*P*<0.01; Figure 2a). In marked contrast, RdB-, RdB/IL12-, RdB/IL23-, RdB/IL23/p35-treated tumors reached to an average volume of 2586.9±405.5 mm^3^, 290.7±32.4 mm^3^, 623.3±83.3 mm^3^, and 79.2±19.6 mm^3^ by 9 days, respectively, demonstrating the superior antitumor efficacy of RdB/IL23/p35. Moreover, all animals that received RdB/IL23/p35 were still viable 30 days after initial treatment (*P*<0.01; Figure 2b), whereas only 25% of RdB/IL12-treated mice were viable and no animal in RdB/IL23 group was viable in the same time period (*P*<0.01; Figure 2b).

It may be particularly important to test the therapeutic effect on large tumors that is commonly observed clinically. Therefore, we further evaluated the antitumor efficacy of RdB/IL23/p35 on large tumors burden. Mice were treated with PBS, RdB, dE1/IL23/p35 (replication-incompetent Ad co-expressing IL-23 and p35), or RdB/IL23/p35 (oncolytic Ad co-expressing IL-23 and p35) when initial tumor reached an average volume of 500 mm^3^. As presented in Figure 2c and 2d, the antitumor effect and survival rate were markedly enhanced in large tumors treated with RdB/IL23/p35 as compared with large tumors treated with RdB (*P*<0.01), resulting in complete regression in all 7 mice treated. More importantly, RdB/IL23/p35 exhibited a greater antitumor effect as well as prolonged survival than dE1/IL23/p35 (*P*<0.01), thus illustrating the contribution of oncolytic Ads in therapeutic benefit of RdB/IL23/p35. Taken together, these results suggest that RdB/IL23/p35 can lead to potent antitumor activity even in large tumors masses representative of late-stage solid tumors.

### In vivo oncolytic Ad treatment increased local expression of IL-12, IL-23, IFN-γ, and TNF-αin tumor tissues

**Figure 3:**
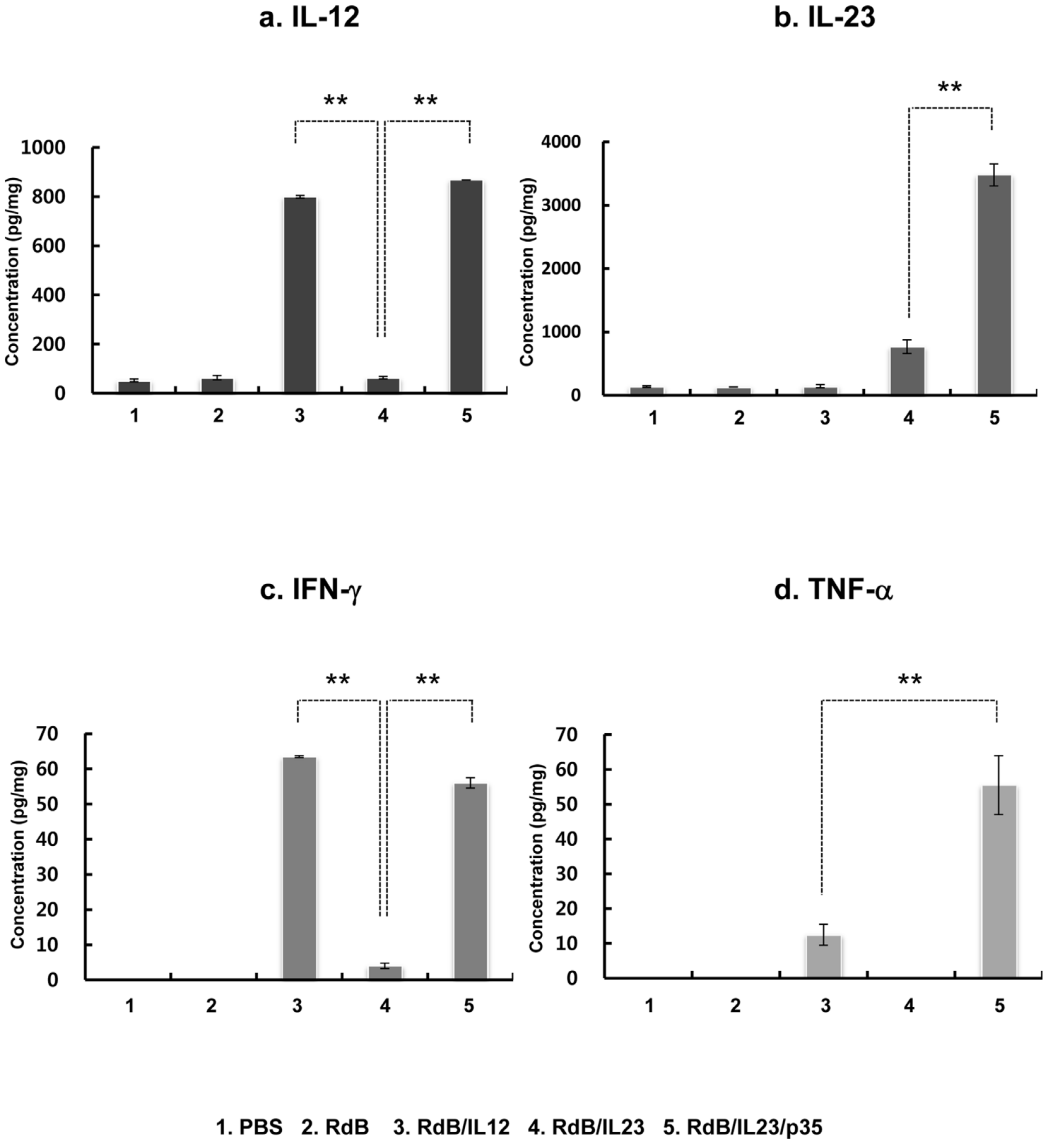
Local expression of IL-12, IL-23, IFN-γ, and TNF-α *in vivo*. Tumor tissue was obtained at 7 days after the first viral treatment. ELISA was performed to estimate the level of IL-12 (a), IL-23 (b), IFN-γ (c), and TNF-α (d) in tumor tissues. Experiments were carried out in triplicate and repeated three times. Each data point indicates means ± SE of IL-12, IL-23, IFN-γ, and TNF-α levels for each individual tumor. **, P<0.01 compared with RdB/IL12 or RdB/IL23 alone.

To determine the levels of IL-12 and IL-23 expressed in RdB/IL12-, RdB/IL23-, or RdB/IL23/p35-administrated animals, tumor tissues were harvested 3 days after the final viral injection. As shown in Figure 3a, tumors treated with RdB/IL12 or RdB/IL23/p35 showed high concentrations of IL-12 (799.2±6.6 pg/mg for RdB/IL12 and 867.8±0.0 pg/mg for RdB/IL23/p35) (*P*<0.01), whereas a minimal amount of IL-12 was observed in the tumors treated with PBS, RdB, or RdB/IL23 (Figure 3a). In addition, RdB/IL23/p35-inoculated tumors showed the highest IL-23 expression (3334.0±51.5 pg/mg) compared to the other tumors treated with PBS (128.5±4.8 pg/mg), RdB (137.0±3.8 pg/mg), RdB/IL12 (167.3±3.3 pg/mg), or RdB/IL23 (862.3±11.0 pg/mg) (*P*<0.01; Figure 3b).

Since IL-12 and IL-23 can stimulate the production of IFN-γ and TNF-αfrom T and NK cells [8], [19], [20], we further assessed the expression levels of IFN-γ and TNF-α in the tumor tissues. As shown in Figure 3c and d, RdB/IL12- or RdB/IL23/p35-received groups exhibited high levels of IFN-γ (2364.4±265.4 pg/mg for RdB/IL12 and 3590.6±213.5 pg/mg for RdB/IL23/p35) (*P*<0.01), whereas PBS-, RdB-, and RdB/IL23-received groups exhibited relatively low levels of IFN-γ(342.6±54.6, 179.2±80.7, and 391.2±159.7 pg/mg, respectively). Moreover, RdB/IL23/p35-inoculated tumors showed the highest TNF-α expression (27.7±4.7 pg/mg) compared to the other tumors treated with PBS (0.0±0.0 pg/mg), RdB (0.0±0.0 pg/mg), RdB/IL12 (12.5±3.0 pg/mg), and RdB/IL23 (0.0±0.0 pg/mg) (*P*<0.01; Figure 3d). Taken collectively, these data demonstrate that RdB/IL23/p35-treated tumors exhibit simultaneous high expression of Th1 cytokines such as, IL-12, IL-23, IFN-γ, and TNF-α, suggesting that RdB/IL23/p35 can shift T cell responses toward the type 1 pattern by enhancing Th1 cytokine in the tumor milieu, leading to enhanced antitumor immunity.

### Generation of tumor-specific immune cells

**Figure 4:**
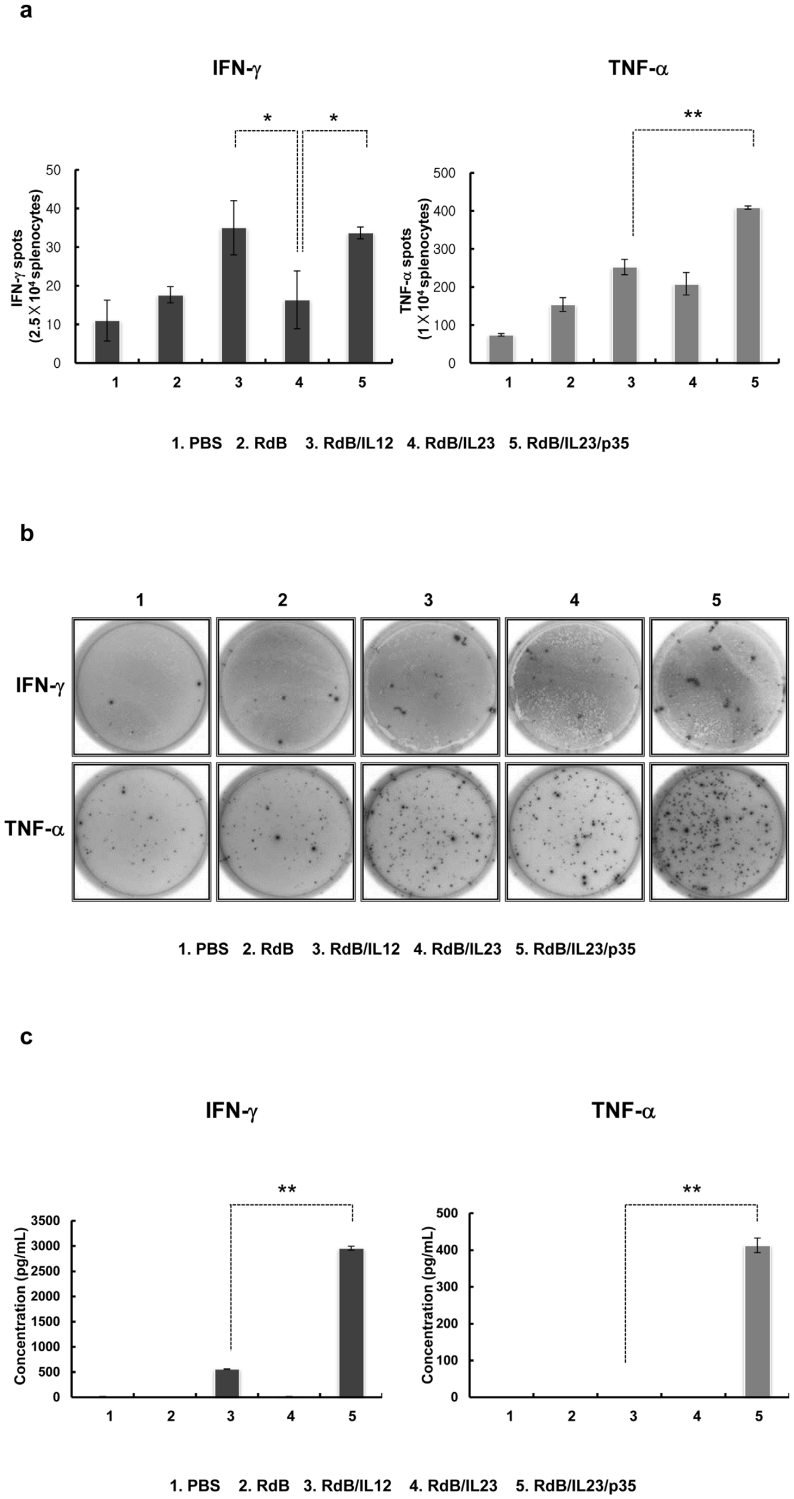
Assessment of tumor-specific immunity. Splenocytes were collected from PBS-, RdB-, or cytokine-expressing oncolytic Ads-treated mice at 7 days following the first viral treatment, and co-incubated with pre-irradiated B16-F10 cells for 1 day. IFN-γ and TNF-α ELISPOT assays were then carried out. (a) The number of spots counted at a concentration of 2.5×10^5^ (IFN-γ ELISPOT) or 1×10^4^ (TNF-α ELISPOT). Each value represents the mean spot number ± SE of triplicates of representative of three independent experiments. *, P<0.05 or **, P<0.01 compared with RdB/IL12 or RdB/IL23. (b) Examples of spot-forming cell (SFC) response. (c) Quantification of IFN-γ and TNF-α released by tumor-specific immune cells. Splenocytes were collected from PBS-, RdB-, or cytokine-expressing oncolytic Ads-treated mice, and co-incubated with pre-irradiated B16-F10 cells for 3 days. The concentration of IFN-γ and TNF-α was then evaluated in the co-cultured supernatants by ELISA kit. Data represent the mean ± SE of triplicate experiments. **, *P*<0.01 compared with RdB/IL12.

RdB/IL23/p35 might create a tumor milieu that is more favorable to induce tumor-specific immunity than an oncolytic Ad expressing either IL-12 or IL-23 alone. Therefore, we further investigated the level of tumor-specific immune response by conducting IFN-γ or TNF-α ELISPOT assay. Spleen cells from mice at 3 days after the last viral administration were harvested and co-cultured with irradiated B16-F10 or NIH3T3 for 24 hr in the presence of recombinant human IL-2. The co-cultured splenocytes were then assessed for IFN-γ- or TNF-α-secreting immune cells via IFN-γ or TNF-α ELISPOT kit. As presented in Figure 4a, the population of IFN-γ-producing immune cells recovered from mice injected with RdB/IL12 or RdB/IL23/p35 was remarkably greater than cells from mice with one of the other Ads. Since each spot reflects the activity of a single cell, and the spot size correlates with the amount of cytokine secreted per cell analyzed in ELISPOT assay [21], we further evaluated the spot size distribution of IFN-γ-secreting immune cells. Interestingly, we observed that spot size of immune cells secreting IFN-γ were significantly increased in the mice treated with RdB/IL23/p35 compared with RdB/IL12-received mice (Figure 4b), even though the number of IFN-γ-producing immune cells is not differ between RdB/IL12 and RdB/IL23/p35. These findings suggest that RdB/IL23/p35 elicits remarkable elevation in per-cell IFN-γ, rather than an increase of IFN-γ-secreting immune cells frequencies compared with RdB/IL12. In addition, we observed a significant increase in the spot size as well as the number of TNF-α-secreting immune cell in mice treated with RdB/IL23/p35 compared with RdB/IL12- or RdB/IL23-inoculated animals (Figure 4a and 4b; *P*<0.01). Further, no significant differences in the number of immune cells producing IFN-γ or TNF-α were observed when NIH3T3 cells were used as a specificity control (Figure S2a). Taken together, these data suggest that IL-12 more effectively induced Th1 immune responses over IL-23 alone, and this response was significantly enhanced when combined with IL-23.

To further confirm whether RdB/IL23/p35 mediates up-regulation of antitumor cytokines (IFN-γ and TNF-α) secreted by tumor-specific immune cells, we co-cultured splenocytes with irradiated B16-F10 or NIH3T3 for 3 days in the presence of recombinant human IL-2. The culture supernatants were then analyzed for IFN-γ and TNF-α production using ELISAs. As seen in Figure 4c, we observed a significant elevation in the IFN-γ and TNF-α production of tumor-specific immune cells in groups administrated with RdB/IL23/p35 compared with RdB/IL12-administrated groups (*P*<0.01), confirming the results obtained by IFN-γ and TNF-α ELISPOT assay. No significant elevation in the IFN-γ and TNF-α secretion of immune cells was observed when NIH3T3 cells were used as a specificity control (Figure S2b). Taken together, these observations demonstrate that animals treated with RdB/IL23/p35 had a higher tumor-specific adaptive immunity than those treated with RdB/IL12 or RdB/IL23.

### The increased population of IFN-γ- and TNF-α-co-producing T cells in DLNs

**Figure 5:**
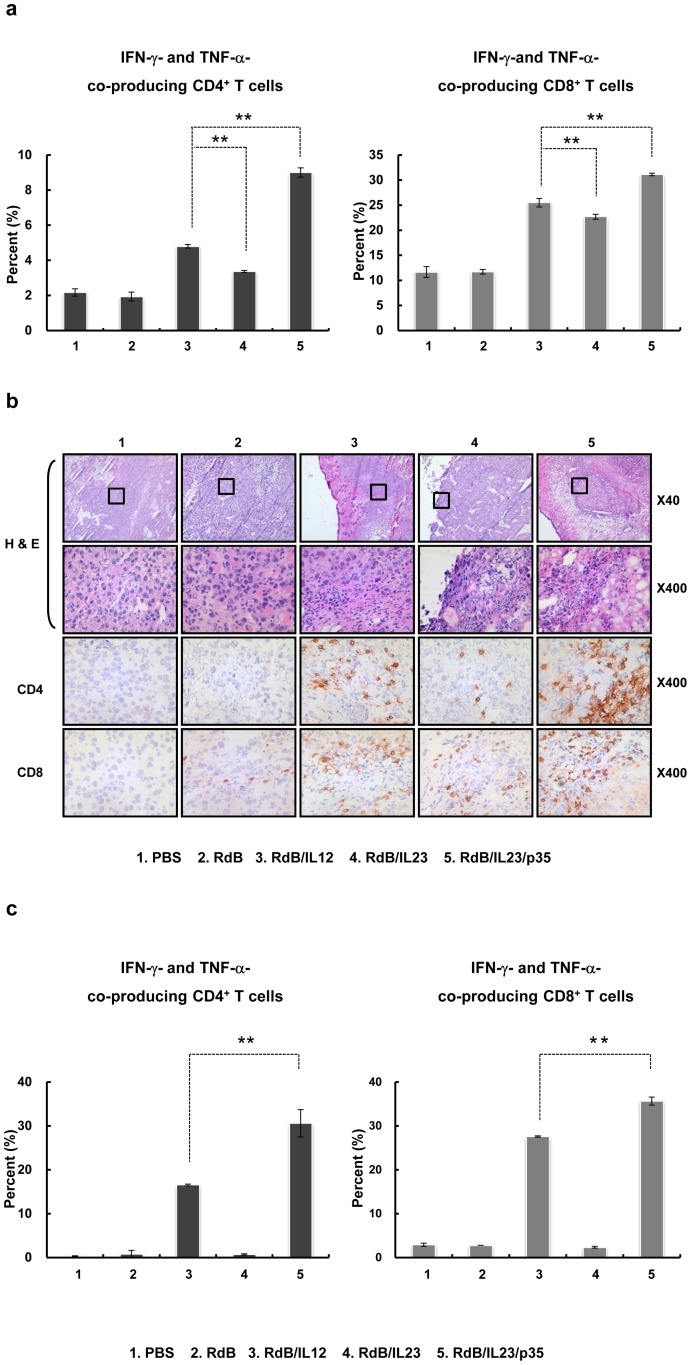
The increased frequency and tumor infiltration of IFN-γ- and TNF-α-co-expressing T cells in RdB/IL23/p35-treated mice. DLNs (a) and tumor-infiltrating lymphocytes (c) were collected from mice treated with PBS, RdB, or cytokine-expressing oncolytic Ads at 7 days after the first viral treatment, and were analyzed after gating on the mononuclear lymphocyte population. CD4^+^ or CD8^+^ T cells were gated on and analyzed for IFN-γ^+^ and TNF-α^+^ cells. **, P<0.01 compared with RdB/IL12 or RdB/IL23 alone. (b) Histological and immunohischemical analysis of tumor sections from mice treated with Ads. Ads were injected on days 0, 2, and 4, and tumors were collected on day 7 for histological analysis. Frozen sections of tumor tissue were then stained by H & E. (top two rows, Original magnification: ×40 & ×400). Cryo-sections of tumor tissue were stained with anti-CD4 (third row) and anti-CD8 (fourth row) monoclonal antibodies. Original magnification: ×400.

Recently, new evidences have indicated that antigen-specific T cells include subsets of T cells that express different combinations of two cytokines, and that the relative contributions of these populations was highly relevant for cell-mediated effector functions and clinical outcome [22], [23]. Accordingly, we investigated the frequency of IFN-γ- and TNF-α-co-expressing T cells in DLNs to verify the therapeutic mechanism of antitumor immunity induced by RdB/IL23/p35. As presented in Figure 5a, subsets of IFN-γ- and TNF-α-co-expressing CD4^+^/CD8^+^ T cells were dramatically elevated in the mice treated with RdB/IL23/p35 compared with RdB/IL12- or RdB/IL23-treated groups (*P*<0.01), indicating that the strongest Th1 response was induced in mice treated with RdB/IL23/p35. Furthermore, the populations of IFN-γ- and TNF-α-co-expressing T cells in the RdB/IL12-received hosts was increased over the RdB/IL23-received hosts (*P*<0.01), which is consistent with a better antitumor response (Figure 2). Taken together, IL-12 effectively promotes the generation of tumor-specific immune cells co-producing IFN-γ and TNF-α and the response is markedly enhanced when IL-23 is combined with IL-12.

### The increased infiltration of IFN-γ- and TNF-α-co-producing T cells in RdB/IL23/p35-treated tumor

To investigate whether lymphocytes infiltrated into tumor tissues, we performed histologic analysis with H & E staining. As seen in Figure 5b, in the RdB/IL23/p35-administrated groups, almost all of the tumor tissues were observed massive lymphatic infiltration as well as necrotic region compared with RdB/IL12- or RdB/IL23-treated tumor tissues. Further, denser immune cell infiltration was observed not only around, but also inside the remaining tumor tissues treated with RdB/IL23/p35. Moreover, to identify the cells infiltrated into the tumor tissues, tumor sections were examined by immunohistochemistry using anti-CD4 or anti-CD8 monoclonal antibodies. Higher frequencies of CD4^+^ and CD8^+^ T cells were observed in both the center and border zones of the tumors treated with RdB/IL23/p35 when compared with RdB/IL12- or RdB/IL23-treated tumor tissues (Figure 5b and Figure S1).

Importantly, to investigate whether CD4^+^ and CD8^+^ T cells infiltrated in RdB/IL23/p35-infected tumor tissues are IFN-γ- and TNF-α-co-expressing T cells, FACS analysis was carried out on lymphocytes isolated from tumor. As shown in Figure 5c, the IFN-γ- and TNF-α-co-secreting T cell population was dramatically greater in tumor tissues treated with RdB/IL23/p35 than those in tumors treated with either RdB/IL12 or RdB/IL23 alone. These findings indicate that a potent antitumor immune response can be induced in the tumor tissue by intratumoral injection of oncolytic Ad co-expressing IL-23 and p35. Additionally, the therapeutic mechanism of this antitumor immunity in mice inoculated with RdB/IL23/p35 is associated with the generation and infiltration of IFN-γ- and TNF-α-co-secreting T cells in tumor microenvironment.

### The reduction of Treg cells in RdB/IL23/p35-treated mice

**Figure 6:**
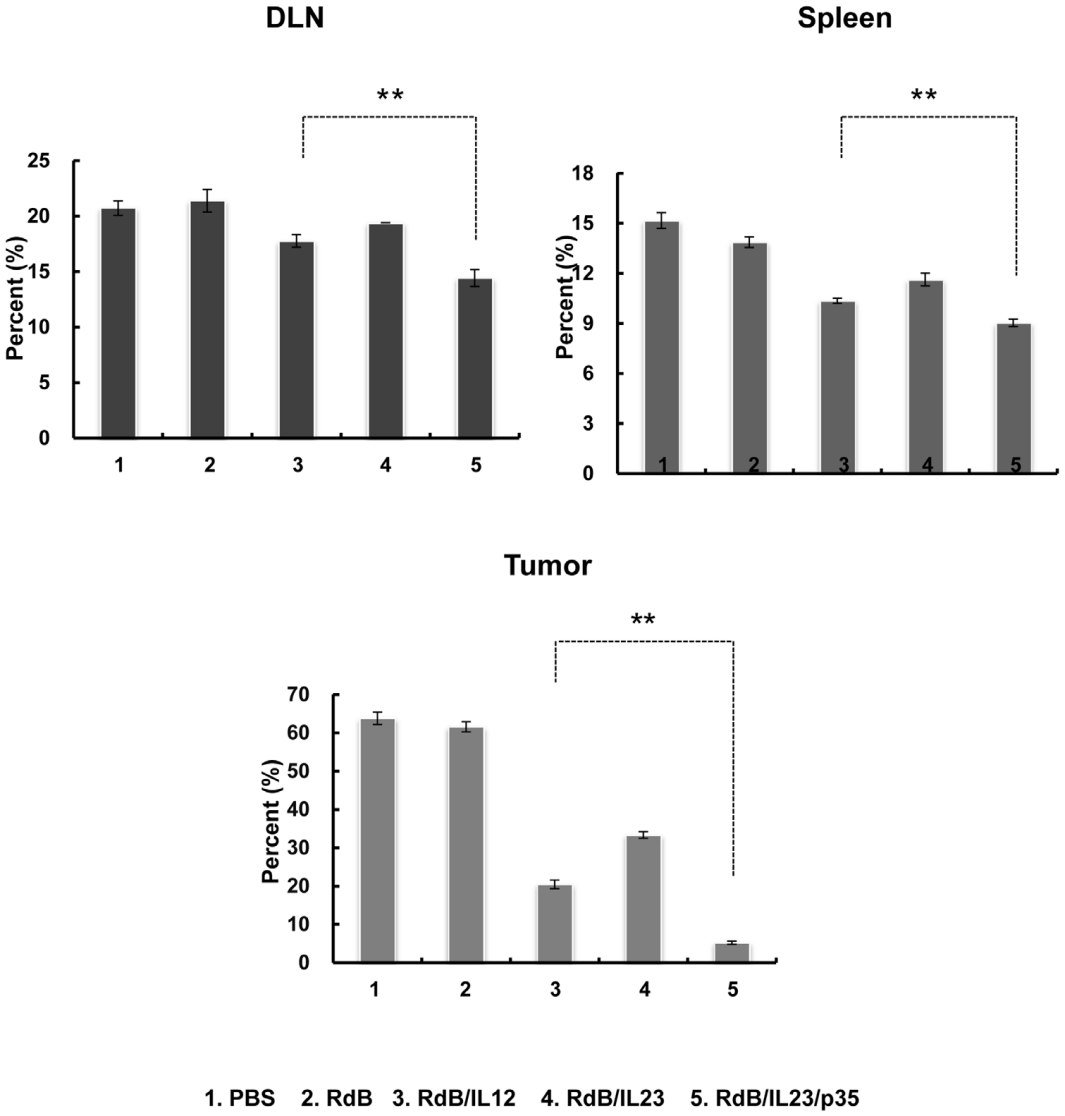
The frequency of Treg cells in DLNs, spleens, and tumors. DLNs, spleens, and tumors were collected from mice treated with PBS, RdB, or cytokine-expressing oncolytic Ads at 7 days following the first viral treatment, and were analyzed after gating on the mononuclear lymphocyte population. CD4^+^ T cells were gated on and analyzed for both CD25^+^ and Foxp3^+^ cells. Each data point indicates mean ± SE of triplicates of representative of three independent experiments. **, P<0.01 compared with RdB/IL12.

IFN-γ and TNF-αhas been shown to mediate inhibition of Treg cell proliferation [24], [25]. As described above, IFN-γ and TNF-αlevels produced by T cells were markedly increased in hosts treated with RdB/IL23/p35 (Figure 4). To evaluate changes in the Treg cell population in Ads-administrated animals, therefore, cells were prepared from DLN, spleen, and tumor mass. As presented in Figure 6, Treg cell population from the DLN, spleen, and tumor was significantly decreased in mice treated with RdB/IL23/p35 as compared with mice treated with either RdB/IL12 or RdB/IL23 (*P*<0.01), suggesting that increased number of IFN-γ- and TNF-α-co-secreting T cells in RdB/IL23/p35-administraed mice may contribute to the reduction of Treg cell population.

### Roles of T cells, IFN-γ, and TNF-α in RdB/IL23/p35-mediated antitumor immunity

**Figure 7:**
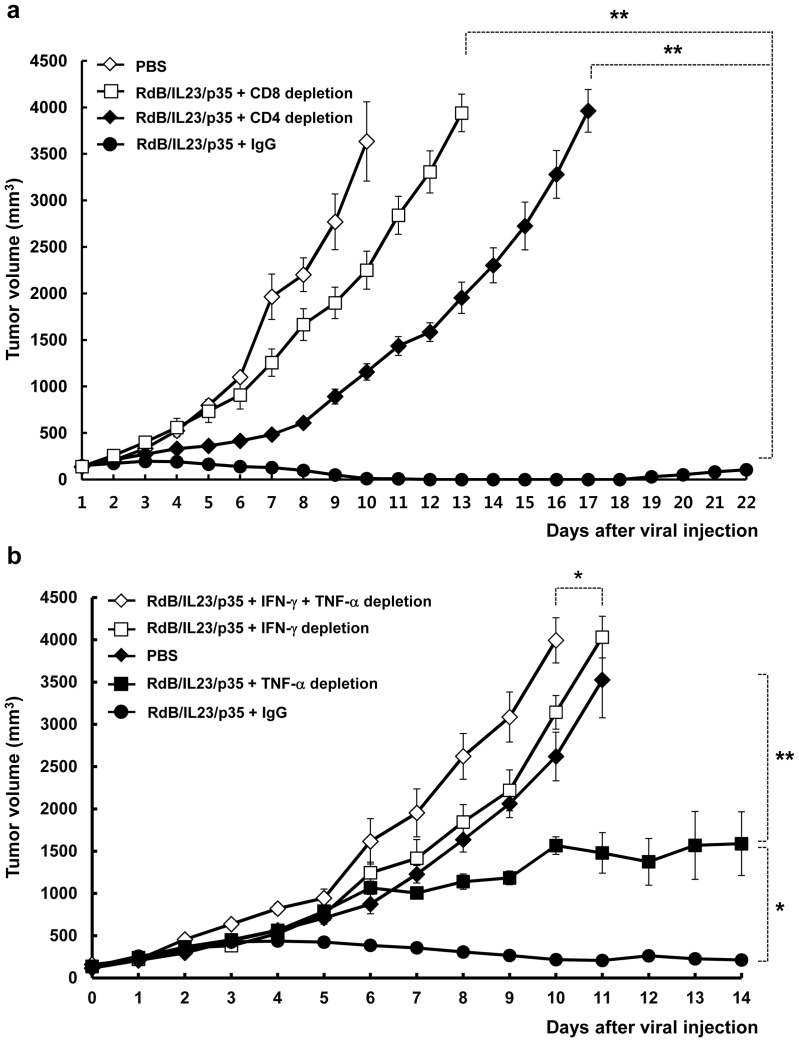
*In vivo* CD4^+^/CD8^+^ T cells or antitumor cytokines (IFN-γ and/or TNF-α depletion in RdB/IL23/p35-administrated mice. C57BL/6 tumor-bearing mice were treated with intratumoral injections of RdB/IL23/p35 and intravenous injection of antibodies [anti-CD4 or anti-CD8 antibodies (a) anti-IFN-γ and/or anti-TNF-αantibodies (b)] according to the protocol described in the Materials and Methods. The control group received isotype-matched antibody (IgG). Tumor growth was monitored and recorded every day. Values represent the mean ± SE (6∼8 animals per group). *, P<0.05; **, P<0.01.

To assess the roles of CD4^+^ and CD8^+^ T cells in RdB/IL23/p35-mediated antitumor immunity, *in vivo* T cell depletion studies were conducted with anti-CD4 or anti-CD8 antibodies. The selective depletion of CD4^+^ or CD8^+^ T cells led to a marked elevation in tumor volume compared with normal IgG-treated mice (*P*<0.01; Figure 7a), suggesting that RdB/IL23/p35 induces both CD4^+^ and CD8^+^ T cells-mediated antitumor immune response.

To further verify the mechanism of RdB/IL23/p35-mediated therapeutic benefit *in vivo*, IFN-γ and/or TNF-α depletion experiments were performed by injecting B16-F10 melanoma-bearing mice with anti-IFN-γ and/or anti-TNF-α antibodies. As seen in Figure 7b, administration of anti-TNF-α partially abrogated the antitumor activity mediated by the RdB/IL23/p35, whereas anti-IFN-γ completely abolished the tumor regression response. Importantly, the loss of both IFN-γ and TNF-α resulted in a significant increase in tumor volume compared with PBS-treated mice (*P*<0.05; Figure 7b), suggesting that both IFN-γ and TNF-αsecreted by T cells are essential and are associated with the therapeutic efficacy of intratumoral injections with RdB/IL23/p35.

## Discussion

The immune escape mechanism within the neoplastic microenvironment is a crucial issue for cancer immunotherapeutic success [5]. The aberrant polarization from the Th1 to Th2 cytokine phenotype, which favors development and progression of cancers, has been reported in a variety of tumor-bearing hosts, and a correlation between Th1 cytokine profile and therapeutic benefit to cancer patients has also been reported [26], [27]. In addition, tumor-associated Treg cells have an important role in suppressing tumor-specific immunity via potential multiple suppressive mechanisms, ultimately enabling the tumor cells to uncontrollably proliferate in the host [28]. Therefore, it is critical to manipulate the tumor milieu more favorable to induce optimal generation of antitumor adaptive immunity in cancer-bearing hosts by overcoming tumor-induced immune suppression.

IL-12 boosts strong antitumor immunity by promoting Th1 differentiation of CD4^+^ T cells and stimulating CTLs and NK cells cytotoxicity [8], [9]. IL-23 is mainly produced by APCs and type 1 macrophages as IL-12, and the receptor complex is expressed or up-regulated on T and NK cells [9], [29]. This IL-23 has been proposed to have similar but not overlapping functions with IL-12 in inducing Th1 cell differentiation and antitumor immune response [13]. Accordingly, in combination gene therapy, local expression of IL-12 and IL-23 may function to induce a potent Th1 immunity and T cell-mediated antitumor immune response, respectively. However, the effects and the underlying mechanisms of local, intratumoral co-expression of IL-12 and IL-23 in a therapeutic cancer model have yet to be investigated.

For simultaneous expression of IL-12 and IL-23 within the tumor microenvironment, we first constructed an oncolytic Ad expressing IL-23 and p35, and showed that RdB/IL23/p35 elicited significantly enhanced IL-12 and IL-23 secretion level compared with that of RdB/IL12 or RdB/IL23 (Figure 1). These observations are significant because previous studies have reported the cytokine gene insertion in both E1 and E3 region of Ad resulted in decreased cytokine expression compared with that in the E1 or E3 region alone [30], [31]. Moreover, we observed that the expression levels of IL-12 and IL-23 mediated by RdB/IL23/p35 was significantly higher than those mediated by RdB/IL23 plus RdB/p35 infection (Figure 1d), suggesting that up-regulation of IL-12 and IL-23 induced by RdB/IL23/p35 is not influenced by p40 gene dose. These results suggest that insertion of an additional transgene in the E1 or E3 region of Ad genome may positively affect the expression of the transgene in the other region, eliciting high expression of IL-12 and IL-23. In earlier study, it has been speculated that viral sequences both upstream and downstream from the additional transgene cassette may contribute to enhanced expression of the transgene in the other region [32]. However, the exact mechanism underlying these observations is unknown. Currently, further investigation of possible mechanisms of this observed higher expression of cytokines is under way.

We next investigated the potential therapeutic benefit of an oncolytic Ad co-expressing IL-23 and p35, and observed that RdB/IL23/p35 elicited not only improved suppression of tumor growth, but also increased survival over either RdB/IL12 or RdB/IL23 (Figure 2a and b). In accordance with these result, histological study revealed immense necrotic regions into the tumor tissues of RdB/IL23/p35-treated groups compared with RdB/IL12- or RdB/IL23-treated groups (Figure 5b). Moreover, higher infiltration of CD4^+^ and CD8^+^ T cells were observed in the tumors injected with RdB/IL23/p35 compared with RdB/IL12- or RdB/IL23-treated tumors (Figure 5b and Supplementary Figure 1). Taken together, these results suggest that the potent antitumor effect may be elicited by a CD4^+^ and CD8^+^ T cell-mediated immune response when IL-12 and IL-23 is locally expressed in combination, compared with either cytokine alone.

To better understand the underlying mechanism of this enhanced RdB/IL23/p35-mediated antitumor effect, we examined the characteristics of RdB/IL23/p35-administrated tumor tissues. The expression level of IL-12 was significantly elevated in tumor tissues treated with RdB/IL12 and RdB/IL23/p35 (Figure 3a). The IL-12 is known to stimulate IFN-γ production from activated T and NK cells [33]. In agreement with these previous findings, we observed significant expression levels of IFN-γ Th1 cytokine, in RdB/IL12- and RdB/IL23/p35-treated mice (Figure 3c). IFN-γ has an important role in inducing cell-mediated antitumor immunity by prompting NK cells, CTLs, and Th1 cells as well as increasing cancer cell immunogenicity through enhancing antigen presentation [34].

IL-23 expression was also increased in tumor tissues treated with RdB/IL23 and RdB/IL23/p35 (Figure 3b). In addition, we observed that RdB/IL23/p35-treated tumors produced significantly higher levels of IL-23 than did those treated with RdB/IL23. IL-12 and IL-23 has been shown to exert TNF-αsecretion by activated T and NK cells [8], [19], [20]. TNF-α in turn boosts DC maturation and macrophages activation, thereby allowing the increase of IL-23 expression in those cells [35]. This positive feedback loop could explain the up-regulation of IL-23 in RdB/IL23/p35-treated tumor tissues. Furthermore, matured DCs and activated macrophages can themselves produce TNF-αas well as IL-23 [13], [36]. In accordance with this positive feedback loop, we found that expression levels of TNF-αwere considerably higher in RdB/IL23/p35-treated mice than in RdB/IL12- or RdB/IL23-treated mice (Figure 3d). TNF-α, Th1 cytokine, has a significant role in promoting tumor-specific immune response via enhancement of CTL development and cytotoxicity as well as antigen presentation [37], [38]. Taken collectively, our findings suggest that RdB/IL23/p35 may create a tumor microenvironment more favorable to activate tumor-specific immune cells via simultaneous high expression of IL-12, IL-23, IFN-γ, and TNF-αin the immunosuppressive microenvironment of tumor.

Multiple studies have provided substantial evidence that the increased frequency of Treg cells occur in patients with malignant tumors and was closely correlated to the poor survival of cancer patients [39], [40]. The Treg cells are dominantly responsible for the immunosuppression in tumor immunity, resulting in impaired immune responses in the cancer-bearing hosts [28]. Therefore, depletion of Treg cells in tumor-bearing host is critical for activating a targeted immune response against the cancer. Here, the Treg cell populations were markedly reduced in the tumor milieu as well as DLN and spleen of hosts treated with RdB/IL23/p35 compared with RdB/IL-12- or RdB/IL23-treated groups (Figure 6). These data suggest that local co-expression of IL-12 and IL-23 may play a critical role in the decrease of Treg cell population in tumor-bearing mice. Thus, reduction of Treg cell frequency via RdB/IL23/p35 might contribute to the reduction of immunosuppression in the tumor-bearing hosts, thereby allowing improved antitumor immunity.

The simultaneous up-regulation of Th1 cytokines in tumor microenvironment and significant decrease of Treg subsets mediated by RdB/IL23/p35 may have a substantial influence on optimal generation of antitumor adaptive immune response. In this study, we demonstrated that mice injected with RdB/IL23/p35 had significantly elevated secretion of IFN-γ or TNF-αin tumor-specific immune cells, than did those given RdB/IL12 or RdB/IL23 (Figure 5c). These observations are further supported by IFN-γ and TNF-α ELISPOT assay that RdB/IL23/p35 elicited a remarkably increased spot size for IFN-γ- or TNF-α-expressing immune cells (Figure 5b). However, the frequency of tumor-specific IFN-γ-secreting immune cells did not differ between RdB/IL12 and RdB/IL23/p35, suggesting that IL-12 effectively induces the generation of tumor-specific immune cells, and the response is markedly enhanced when IL-23 is combined with IL-12. Taken together, these results indicate that RdB/IL23/p35-administrated mice had a stronger tumor-specific adaptive immunity than did RdB/IL12- or RdB/IL23-administrated mice. However, we cannot rule out the potential contribution of viral vector-induced immunity for this potent antitumor immune response elicited by RdB/IL23/p35, thus additional studies are underway in our laboratory to address possible attribution of viral-specific immunity.

The quantitative measurement of IFN-γ-producing T cells has been the most mainly used as an important parameter to evaluate cell-mediated immune response against intracellular pathogens [41], since IFN-γ has been demonstrated to play an important role in mediating the killing effect of a variety of intracellular infectious viruses, bacteria, and parasites [42]. However, much of the data have provided significant evidences that the frequency of IFN-γ^+^ T cells is insufficient to illustrate therapeutic mechanism against intracellular pathogens [43]–[45]. In this regard, TNF-αis another important cytokine capable for inducing Th1 immunity against a variety of intracellular infectious viruses, bacteria, and parasites [46]. More importantly, co-production of IFN-γ and TNF-αhas been shown to elicit improved killing effect of intracellular pathogen compared with either cytokine alone [47]. Therefore, we hypothesize that antitumor immunity mediated by RdB/IL23/p35 results from enhanced generation of multifunctional T cells that secret IFN-γ and TNF-αtogether. Here, we demonstrated that the frequency of IFN-γ- and TNF-α-co-producing T cells in the RdB/IL23/p35-treated mice was significantly increased over the RdB/IL12- or RdB/IL23-treated groups (Figure 5a). Importantly, massive infiltration of the T cells co-expressing IFN-γ and TNF-αwas observed in tumor tissue treated RdB/IL23/p35 (Figure 5c). These findings are in good agreement with result of antitumor effect (Figure 2), and are further supported by *in vivo* T cells or IFN-γ and TNF-αdepletion assay, showing that the loss of CD4^+^/CD8^+^ T cells or IFN-γ and TNF-αresulted in a significant increase in tumor volume compared with normal IgG-treated mice (Figure 7). Taken together, these findings suggest that the therapeutic mechanism of antitumor immune mediated by RdB/IL23/p35 is associated with the generation and recruitment of IFN-γ- and TNF-α-co-expressing T cells in tumor microenvironment.

Oncolytic adenoviral vectors have been widely employed in cancer gene therapy [30], [48]–[50]. In contrast to a replication-incompetent Ad, oncolytic Ad can specifically target and replicate in cancer cells while sparing normal cells, so that it can destroy the tumor cells as well as amplify expression of therapeutic genes through continuous viral replication in neoplastic cells. Especially, oncolytic Ad expressing immunostimulatory genes would exhibit substantially greater antitumor effect than non-replicating Ad expressing immunostimulatory genes. The rationale behind this hypothesis is that oncolytic Ad encoding immunostimulatory genes can elicit not only release of tumor-associated antigens via oncolytic Ad-mediated oncolysis but also sustained production of therapeutic level of immunostimulatory genes long enough to generate antitumor immunity effectively in tumor milieu, resulting in enhancement and prolongation of antitumor activity compared with treatment of replication-incompetent Ad. This hypothesis has been confirmed in this study that RdB/IL23/p35 (oncolytic Ad co-expressing both IL-23 and p35) resulted in enhanced antitumor effect and survival rate compared with dE1/IL23/p35 (replication-incompetent Ad co-expressing IL-23 and p35) (Figure 2c and 2d). These data is further supported by another study that intratumoral injection with MIP-1α- and Ad-FLT-3L-encoding oncolytic Ad, compared with MIP-1α- and Ad-FLT-3L-encoding replication-incompetent Ad, elicits improved antitumor immune response, implying the pivotal contribution of the oncolytic Ads for tumor-specific immunity [51]. Taken together, these data indicate that oncolytic Ads can be responsible for the enhanced therapeutic antitumor immunity through potent oncolysis and expression of therapeutic genes, in contrast to the replication-incompetent Ads.

In conclusion, the finding in our report demonstrates for the first time the potent antitumor immunity of an oncolytic Ad co-expressing IL-23 and p35 in a murine melanoma model. We also provide the first study where a novel underlying mechanism of combination therapies via IL-12 and IL-23 that promotes generation of T cell co-producing IFN-γ and TNF-α. These findings provide a new insight into therapeutic mechanisms through IL-12 plus IL-23 as well as a potential clinical cancer immunotherapeutic agent for the generation of enhanced antitumor immunity.

## Materials and Methods

### Ethics Statement

Animal handling was conducted in accordance with national and international guidelines, in an animal facility accredited by the Association for Assessment and Accreditation of Laboratory Animal Care (AAALAC). The number of animals used was minimized, and all necessary precautions were taken to mitigate pain or suffering. Protocols were approved by the Institutional Animal Care and Use Committee at Yonsei University health system (2010–0174).

### Cell lines and culture

Dulbecco's modified Eagle's medium (DMEM; Gibco BRL, Grand Island, NY) supplemented with 10% fetal bovine serum (FBS; Gibco BRL), L-glutamine (2 mmol/L), penicillin (100 IU/mL), and streptomycin (50 μg/mL) were used as the culture medium. HEK293 (human embryonic kidney cell line expressing the adenoviral E1 region), U343 (human glioma cell line), B16-F10 (murine melanoma cell line), and NIH3T3 (murine embryonic fibroblast line) were obtained from the American Type Culture Collection (ATCC, Manassas, VA). All cell lines were cultured at 37°C in a humidified atmosphere 5% CO_2_ and 95% air. *Escherichia coli* was multiplied in Luria Bertani medium at 37°C.

### Animal studies

Six to 8-week-old male C57BL/6 mice were purchased from Charles River Laboratories International, Inc. (Wilmington, MA) and maintained in a laminar air flow cabinet under specific pathogen-free environment. All facilities are approved by AAALAC (Association Of Assessment And Accreditation Of Laboratory Animal Care), and all animal experiments were carried out according to institutionally approved protocols at Yonsei University College of Medicine (Seoul, Korea).

### Generation of oncolytic Ad expressing IL-23 and p35

To generate oncolytic Ad expressing murine IL-23 composed of p19 and p40, we first amplified p19 gene by reverse transcriptase-polymerase chain reaction (RT-PCR) of total RNA purified from bone marrow of C57BL/6 mice. The resulting 590-bp PCR product was digested with *Xho*I and *EcoR*I and subsequently ligated onto *Xho*I and *EcoR*I site of the pCA14, creating a pCA14-p19. The nucleotide sequence of the amplified PCR product was verified using an ABI PRISM377 automatic DNA sequencer. The IRES region was excised from pcDNA3.1-IRES using *EcoR*I and subcloned into pCA14-p19, generating a pCA14-p19/IRES. *SnaB*I-*Sal*I fragments containing p19/IRES gene was then excised from pCA14-p19/IRES, and subcloned into pRdB E1 shuttle vector [52], resulting in a pRdB-p19/IRES shuttle vector. To generate E1 shuttle vector expressing IL-23 gene, the p40 gene was excised from pcDNA3.1-p40 (Cytokine Bank, Chonbuk University, Chunju, South Korea) using *Hind*III and subcloned into pRdB-p19/IRES shuttle vector predigested with *Hind*III, yielding a pRdB-IL23 shuttle vector. The linearized pRdB-IL23 E1 shuttle vector was then co-transformed into *Escherichia coli* BJ5183 along with the *BstB*I-digested pvmdl324BstBI Ad vector for homologous recombination, generating a RdB/IL23 Ad vector.

To construct an oncolytic Ad expressing IL-23 and p35 at the E1 and E3 region, respectively, the linearized pSP72-E3-p35 E3 shuttle vector was then co-transformed into *Escherichia coli* BJ5183 along with the *BstB*I-digested pvmdl324BstBI for homologous recombination, creating a pRdB-p35 Ad vector. The linearized pRd-IL23 E1 shuttle vector was next co-transformed into *Escherichia coli* BJ5183 along with the *Spe*I-digested pRdB-p35 for homologous recombination, generating a RdB/IL23/p35 Ad vector. The construction method of RdB/IL12 Ad vector is described in our previous report [53].

### Quantification of cytokines

Cells were plated onto six-well plates at 3×10^5^ cells/well, and then infected with RdB, RdB/IL12, RdB/IL23, RdB/IL23/p35, RdBIL12 plus RdBIL23, or RdB/IL23 plus RdB/p35 at multiplicity of infections (MOIs) of 0.1–5. At 48 hr after infection, the supernatants were obtained. The level of IL-12 and IL-23 expression were determined by an ELISA according to the manufacturer's instructions (IL-12 ELISA kit: Endogen, Woburn, MA; IL-23 ELISA kit: eBioscience, San Diego, CA).

For the evaluation of cytokines expression in tumor tissue, tumor tissues were collected from mice treated with Ads at 3 days after last viral treatment. Tumor tissues were homogenized (ART-MICCRA D-8; ART moderne Labortechnik, Munchen, Germany) in ice-cold RIPA buffer (Elipis Biotech, Taejeon, South Korea) with a proteinase inhibitor cocktail (Sigma, St. Louis, MO). Homogenates were then centrifuged in a high-speed microcentrifuge for 10 min and determined for total protein content using a BCA protein assay reagent kit (Pierce, Rockford, IL). Levels of IL-12, IL-23, IFN-γ, and TNF-α were measured by conventional ELISA kits (IL-12 ELISA kit: Endogen; IL-23 ELISA kit: eBioscience; IFN-γ ELISA kit: Endogen; TNF-α ELISA kit: R&D Systems, Minneapolis, MN). Each experiment was carried out three times with three replicates in each group. The results were normalized relative to the total protein concentration in each tumor and were calculated as picograms per milligram of total protein.

To measure cytokines released by tumor-specific immune cells, spleens were obtained aseptically from tumor-bearing mice at 3 days following the last viral treatment, and unicellular splenocytes were prepared as previously described [30]. Prepared 1.5×10^6^ splenocytes were co-cultured with 1.5×10^5^ irradiated B16-F10 or NIH3T3 (5,000 rad) for 3 days in the presence of recombinant human IL-2 (100 units/mL, R&D Systems). The culture supernatants were then harvested and analyzed for IFN-γ and TNF-α production using ELISAs (IFN-γ ELISA kit: Endogen; TNF-α ELISA kit: R&D Systems).

### Total RNA isolation, cDNA synthesis, and RT-PCR analysis

Cells seeded on 6-cm dishes were infected with RdB, RdB/IL12, RdB/IL23, RdB/IL23/p35, or RdBIL12 plus RdBIL23 at an MOI of 5. At 2 days post-infection, the cells were collected and total RNA was prepared by the RNeasy mini kit (Qiagen, Valencia, CA) according to the manufacturer's recommendations. For RT-PCR, 1 μg of total RNA was converted to cDNA by treatment with 200 units of M-MLV reverse transcriptase and 500 ng of oligo(dT) primer in 50 m*M* Tris-HCl (pH 8.3), 75 m*M* KCl, 3 m*M* MgCl_2_, 10 m*M* dithiothreitol, and 1 m*M* dNTPs at 37°C for 1 hr. The reaction was stopped by heating at 70°C for 15 min. Two µL of the cDNA mixture was then used for enzymatic amplification. PCR was performed in 50 m*M* KCl, 10 m*M* Tris-HCl (pH 8.3), 1.5 m*M* MgCl_2_, 0.2 m*M* dNTPs, 2.5 units of Taq DNA polymerase, and 0.1 µ*M* each of primers. The sequences of used were as follows: murine p19, sense primer, 5′-cctggctgtgcctaggagta-3′, and antisense primer, 5′-aggctcccctttgaagatg-3′; murine p35, sense primer, 5′-gccagggtcattccagtctc-3′, and antisense primer, 5′-ggcacagggtcatcatcaaa-3′; murine p40, sense primer, 5′-agcagttcccctgactctcg-3′, and antisense primer, 5′-cagggtactcccagctgacc-3′. The amplification was performed in a DNA thermal cycler (model PTC-200; MJ Research, Waltham, MA) under the following condition: denaturation at 94°C for 10 min for the first cycle and for 1 min starting from the second cycle, annealing at 55°C for 1 min, and extension at 72°C for 1 min for 30 repetitive cycles. The reaction was terminated with a final cycle of extension (72°C for 10 min). The PCR products were then separated by 1% agarose gel electrophoresis. Semi-quantitative RT-PCR was performed using human β-actin as an internal control to normalize gene expression for the PCR templates.

### Tumor model and animal study

Tumors were implanted subcutaneously on abdomen of C57BL/6 mice by injecting B16-F10 murine melanoma cells (5×10^5^) in 50 μL of Hank's balanced salt solution (HBSS; Gibco BRL). When tumors reached a range of 130–150 mm^3^ or 500 mm^3^, animals were randomized into groups (PBS, RdB, RdB/IL12, RdB/IL23, dE1/IL23/p35, or RdB/IL23/p35) of 6∼8 animals each, and treatment was initiated. First day of treatment was designated as day 0. Ads or PBS were administered intratumorally (5×10^9^ VP per tumor in 50 μL of PBS or 1×10^10^ VP per tumor in 150 μL of PBS) on days 0, 2, and 4. Tumor growth was monitored by measuring the length (*L*) and width (*w*) of the tumor. Tumor volume was calculated using the following formula: volume  = 0.523*L*(*w*)^2^. Tumor responses to each treatment were compared using a logrank test analysis (StatView software; Abacus Concepts, Inc., Berkeley, CA).

Cancer progression was monitored everyday over a period of 30 days based on overall health until a predetermined endpoint was reached (tumor size>3000 mm^3^). Survival time reflects the time required for the animals to reach humane endpoints, including tumor ulceration, weight loss exceeding 15%, emaciation, anorexia, and/or diarrhea. Survival curve was then plotted against time after treatment.

To neutralize IFN-γ and/or TNF-α *in vivo*, mice were administrated intravenously with 200 μg anti-IFN-γ (clone R4-6A2; BioXCell, West Lebanon, NH) and/or anti-TNF-α (clone XT3.11; BioXCell) neutralizing antibodies 1 day before virus injection and once every 2 days thereafter for an additional 10 days. The control group received isotype-matched antibody (BioXCell). RdB/IL23/p35 was inoculated as described above in “antitumor effect”.

To deplete CD4^+^ or CD8^+^ T cells *in vivo*, mice were inoculated intravenously with 200 μg anti-CD4 (clone GK1.5; DiNonA, Seoul, Korea) or anti-CD8 (clone 53–6.7; DiNonA) neutralizing antibodies 2 days before virus injection and once every 5 days thereafter for an additional 15 days. The control group received isotype-matched antibody (BioXCell). RdB/IL23/p35 was inoculated as described above in “antitumor effect”.

### IFN-γ and TNF-α enzyme-linked immune spot (ELISPOT) assay

To assess the population of antigen-specific cytokine-producing cells, IFN-γ and TNF-α ELISPOT assay were performed. Seven days after the first Ad injection, spleens were collected aseptically from mice bearing a B16-F10 tumor, and unicellular splenocytes were prepared as described above in “Quantification of cytokines”. Prepared spleen cells were co-cultured with pre-irradiated B16-F10 or NIH3T3 (5,000 rad) cells for 24 hr in culture medium supplemented with human IL-2 (100 units/mL, R&D Systems). IFN-γ and TNF-α ELISPOT assay were then carried out according to the manufacturer's specifications (IFN-γ ELISPOT kit: BD Biosciences, San Jose, CA; TNF-α ELISPOT kit: R&D Systems). The colored spots, representing IFN-γ- or TNF-α-producing cells, were counted with a KS-ELISpot (Zeiss-Kontron, Jena, Germany), and confirmed by the computer-based Immunospot system (AID Elispot Reader System, Version 3.4; Autoimmun Diagnostika GmbH, Strassberg, Germany).

### Isolation of lymphocytes from tumor

After removing fat, blood, and necrotic areas, tumor tissues were washed in HBSS (Gibco BRL), cut into small pieces in a petri dish covered with RPMI 1640 medium (Gibco BRL), and dissociated for 2 hr with shaking at 37°C using a mixture of enzymes dissolved in RPMI 1640 medium (Gibco BRL) containing 5% FBS [collagenase (10 mg/mL; Sigma) and DNase I (25 mg/mL; Sigma)]. The digest was then passed through 70-µm cell-strainers (BD Biosciences) to remove clumps and debris, and the filtrate was washed two times in medium followed by centrifugation at 470 g for 5 min. To separate tumor cells from lymphocytes, the cell suspension was layered onto a discontinuous gradient of 75% over 100% Ficoll-Hypaque (GE Healthcare Bio-sciences AB, Uppsala, Sweden) in medium and centrifuged at 800 g for 20 min at room temperature. Lymphocytes were harvested from the interphase between 75% and 100% Ficoll-Hypaque and washed in HBSS (Gibco BRL).

### Fluorescence-activated cell sorting (FACS) analysis

For the assessment of IFN-γ- and TNF-α-co-expressing T cell population, draining lymph nodes (DLNs) and tumor tissues were harvested at 7 days following first viral inoculation of the B16-F10 tumor-bearing mice. DLN cells or lymphocytes isolated from tumor tissue were stimulated for 3 hr with phorbol myristate acetate (50 ng/mL; Sigma) and ionomycin (250 ng/mL; Sigma) in the presence of Golgiplug at the recommended concentrations (BD PharMingen, San Diego, CA). Before staining, cells were treated with saturating anti-CD16/CD32 (Biolegend, San Diego, CA) in staining buffer (2% FBS and 0.02% sodium azide in PBS). Cells were stained extracellularly with fluorescein isothiocyanate (FITC)-conjugated anti-CD4 (eBioscience) and peridinin chlorophyll protein (PerCP)-CY5.5-conjugated anti-CD8 (BD PharMingen), fixed, permeabilized with Cytofix/Cytoperm solution (BD PharMingen), and then stained intracellularly with allophycocyanin (APC)-conjugated anti-IFN-γ (BD PharMingen) and phycoerythrin (PE)-conjugated anti-TNF-α (BD PharMingen). To determine the percentage of IFN-γ- and TNF-α-co-expressing CD4^+^ or CD8^+^ T cell population, lymphocytes were gated by plotting forward vs. side scatter followed by gating on CD4^+^ or CD8^+^ cells. Gated cells were then analyzed for both IFN-γ^+^ and TNF-α^+^cells.

For the assessment of Treg population, DLNs, spleens, and tumors were harvested aseptically from mice at 3 days after the last Ad injection, and were prepared as described above in “Materials and Methods”. After staining of surface markers with PerCP-CY5.5-conjugated anti-CD4 (BD PharMingen) and PE-conjugated anti-CD25 (eBioscience), cells were fixed and permeabilized using the Foxp3 fixation/permeabilization buffer (eBioscience) according to the supplier's protocol. Cells were then stained with APC-conjugated anti-Foxp3 (eBioscience). To determine the percentage of Treg population, lymphocytes were gated by plotting forward vs. side scatter followed by gating on CD4^+^ T cells. Gated cells were then analyzed for both CD25^+^ and Foxp3^+^ cells. Samples were analyzed using a BD Biosciences BD-LSR II Analytic Flow Cytometer and FACSDiva software (BD Biosciences).

### Histology and immunohistochemistry

Tumor tissues for histological study were frozen in OCT compound (Sakura Finetec, Torrance, CA), and cut into 10-μm sections. Representative sections were stained with hematoxylin-eosin (H & E) and then examined by light microscopy. To detect lymphocytes, the cryosections were treated with purified rat anti-mouse CD4 monoclonal antibody (BD PharMingen) or purified rat anti-mouse CD8 monoclonal antibody as a primary antibody, and then with biotin-conjugated goat anti-rat IgG (BD PharMingen) as a secondary antibody. Diaminobenzidine/hydrogen peroxidase (DAKO, Carpinteria, CA) was used as the chromogen substrate. All slides were counterstained with Meyer's hematoxylin.

### Statistical Analysis

The data were expressed as mean ± standard error (SE). Statistical comparison was made using Stat View software (Abacus Concepts, Inc., Berkeley, CA) and the Mann-Whitney test (non-parametric rank sum test). *P* values less than 0.05 were considered statistically significant (*, *P*<0.05; **, *P*<0.01).

## Supporting Information

Figure S1 **Higher frequency of CD4^+^ and CD8^+^ T cells were observed in both center and border zones of the tumors treated with RdB/IL23/p35, in comparison to those treated with either RdB/IL12 or RdB/23.** Ads were injected on days 0, 2, and 4, and tumors were collected on day 7 for histological analysis. Cryo-sections of tumor tissue were stained with anti-CD4 (a) or anti-CD8 (b) monoclonal antibody. Original magnification: ×100.
(TIF)

Figure S2 **Assessment of tumor-specific immunity.** (a) The number of spots counted at a concentration of 2.5×10^5^ (IFN-γ ELISPOT) or 1×10^4^ (TNF-α ELISPOT). Splenocytes were collected from PBS-, RdB-, or cytokine-expressing oncolytic Ads-treated mice at 7 days after the first viral treatment, and co-incubated with pre-irradiated NIH3T3 cells for 24 hr. IFN-γ and TNF-α ELISPOT assays were then carried out. (b) Quantification of IFN-γ and TNF-α released by splenocytes co-cultured with pre-irradiated NIH3T3 for 3 days.
(TIF)
