# Supplementary figures and images for: Oncolytic Adenovirus Expressing IL-23 and p35 Elicits IFN-γ- and TNF-α-Co-Producing T Cell-Mediated Antitumor Immunity
Source: PLoS One. 2013 Jul 3;8(7):e67512. doi: 10.1371/journal.pone.0067512 (PMC3701076; doi:10.1371/journal.pone.0067512)

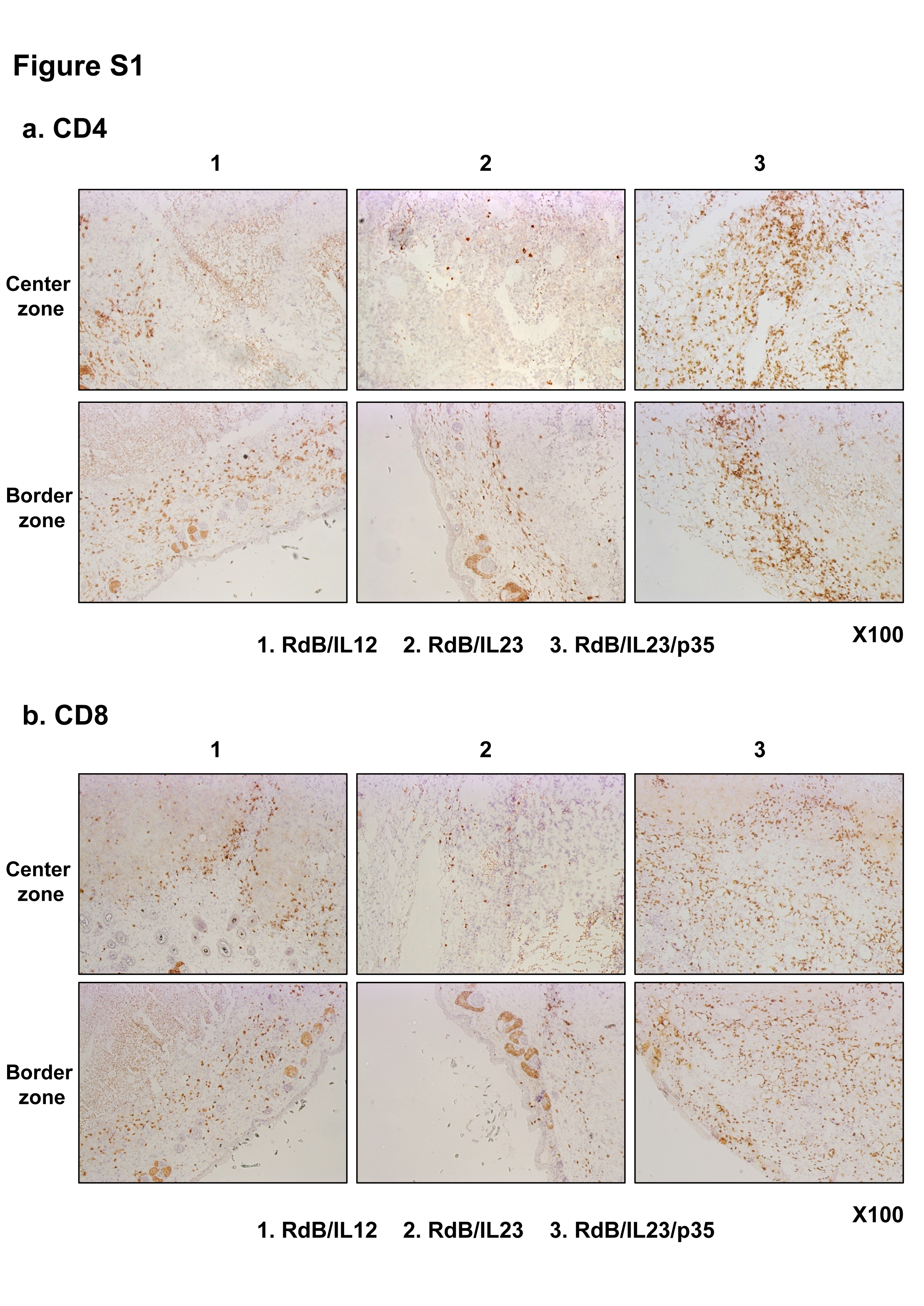

Supplement: Figure S1 — Higher frequency of CD4+ and CD8+ T cells were observed in both center and border zones of the tumors treated with RdB/IL23/p35, in comparison to those treated with either RdB/IL12 or RdB/23. Ads were injected on days 0, 2, and 4, and tumors were collected on day 7 for histological analysis. Cryo-sections of tumor tissue were stained with anti-CD4 (a) or anti-CD8 (b) monoclonal antibody. Original magnification: ×100. (TIF) [file pone.0067512.s001.tif]

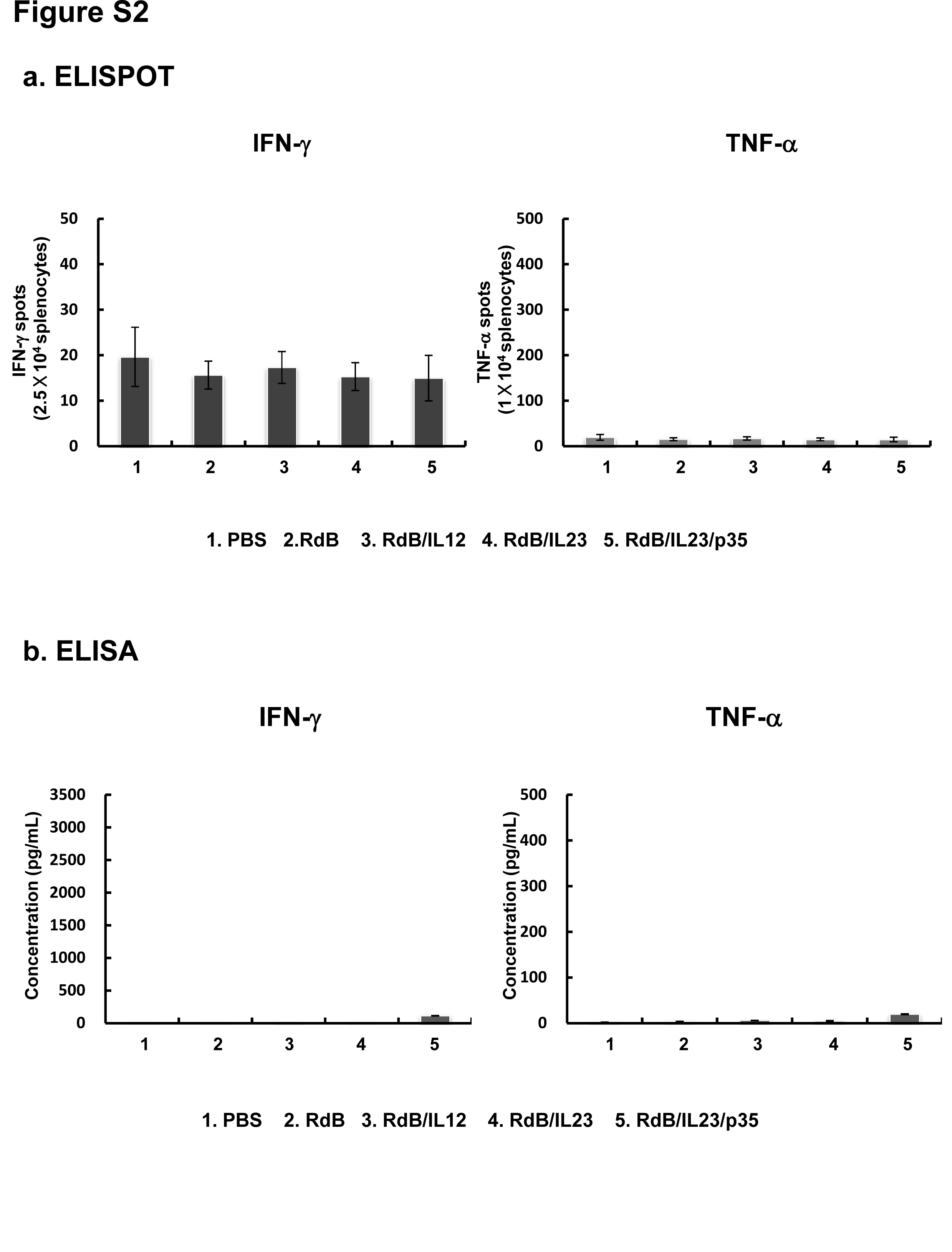

Supplement: Figure S2 — Assessment of tumor-specific immunity. (a) The number of spots counted at a concentration of 2.5×10^5^ (IFN-γ ELISPOT) or 1×10^4^ (TNF-α ELISPOT). Splenocytes were collected from PBS-, RdB-, or cytokine-expressing oncolytic Ads-treated mice at 7 days after the first viral treatment, and co-incubated with pre-irradiated NIH3T3 cells for 24 hr. IFN-γ and TNF-α ELISPOT assays were then carried out. (b) Quantification of IFN-γ and TNF-α released by splenocytes co-cultured with pre-irradiated NIH3T3 for 3 days. (TIF) [file pone.0067512.s002.tif]
